# Disease surveillance based on Internet-based linear models: an Australian case study of previously unmodeled infection diseases

**DOI:** 10.1038/srep38522

**Published:** 2016-12-20

**Authors:** Florian Rohart, Gabriel J. Milinovich, Simon M. R. Avril, Kim-Anh Lê Cao, Shilu Tong, Wenbiao Hu

**Affiliations:** 1 The University of Queensland Diamantina Institute, The University of Queensland, Translational Research Institute, Woolloongabba, 4102, QLD, Australia; 2 School of Medicine, The University of Queensland, St Lucia, 4072, QLD, Australia; 3 School of Public Health and Social Work, Queensland University of Technology, Kelvin Grove, 4056, QLD, Australia; 4 Freelance Developer, The Gap, 4061, QLD, Australia

## Abstract

Effective disease surveillance is critical to the functioning of health systems. Traditional approaches are, however, limited in their ability to deliver timely information. Internet-based surveillance systems are a promising approach that may circumvent many of the limitations of traditional health surveillance systems and provide more intelligence on cases of infection, including cases from those that do not use the healthcare system. Infectious disease surveillance systems built on Internet search metrics have been shown to produce accurate estimates of disease weeks before traditional systems and are an economically attractive approach to surveillance; they are, however, also prone to error under certain circumstances. This study sought to explore previously unmodeled diseases by investigating the link between Google Trends search metrics and Australian weekly notification data. We propose using four alternative disease modelling strategies based on linear models that studied the length of the training period used for model construction, determined the most appropriate lag for search metrics, used wavelet transformation for denoising data and enabled the identification of key search queries for each disease. Out of the twenty-four diseases assessed with Australian data, our nowcasting results highlighted promise for two diseases of international concern, Ross River virus and pneumococcal disease.

Traditional, infectious disease surveillance systems typically rely upon data submitted to public health authorities by medical practitioners, laboratories and other health care providers^1^. These systems are critical to the effective functioning of health systems and form a central component in infectious disease prevention and control. The structure of data collection employed by traditional surveillance systems, however, introduces an inherent lag; the average delay from receipt to dissemination of data by a traditional disease surveillance network is reported to be around two weeks^2^. This lag is created by tardy reporting (or failure to report) and the hierarchical nature of information flow within these systems^3^. Furthermore, resource constraints and a lack of operational knowledge of reporting procedures are recognised to further affect both timeliness and completeness of reporting by traditional surveillance systems^4^; any delay in provision of data may negatively impact the effectiveness of services or may produce an incomplete picture of conditions of interest within the community.

Internet-based surveillance systems have been proposed as a complementary method to collecting information regarding disease in the community that may improve timeliness. A number of approaches to develop Internet-based surveillance systems were published or reviewed^5^,^6^. Briefly, Internet-based surveillance systems attempt to produce a picture of disease in the community through the analysis of Internet-based, health-related information, as well as the distribution and patterns of access of these data^6^. Data sources used for this may include online news stories or case reports, as is used by HealthMap^7^ (http://healthmap.org/); Wikipedia access logs^8^; social media (such as Twitter)^9^,^10^,^11^; or may use participatory approaches (crowdsourcing) for data collection^12^. The majority of work has, however, focused on the use of Internet search metrics^6^,^13^,^14^,^15^. Approaches based on internet search metrics hypothesize that, when people contract a disease, they will search for information on their condition/symptoms on the internet and that accurate estimates of disease occurrence in the community may be produced by monitoring changes in the frequency of specific searches. The best-known examples of this approach were the recently defunct Google Flu Trends^13^,^16^ (http://www.google.org/flutrends/) and Google Dengue Trends^17^ (http://www.google.org/denguetrends/) websites, as well as another approach using search query to infer influenza-like illness rates available online http://fludetector.cs.ucl.ac.uk/ 18. Whilst infectious disease surveillance systems based on search metrics have shown great promise^6^, they have also (rightly) been the target of criticism^19^,^20^,^21^. There are a number of examples where internet-based surveillance systems have provided inaccurate or untimely estimates of disease. As search-query based surveillance systems rely upon health seeking behaviour of the general public, they are heavily influenced by the public’s knowledge, attitudes and behaviour. Whilst there is an increasing body of work describing the relationship between search metrics and disease notification rates, including our own studies^14^,^22^, little work has been done for diseases unrelated to influenza-like illness.

The goal of this study was to assess the potential of internet-based surveillance systems to nowcast previously unmodeled infectious diseases, with varying aetiologies, using Australian data. Current and state-of-the-art modelling methods often require choosing several parameters that are disease-dependent. We propose instead using alternative modelling strategies that take advantage of linear models flexibility to model a wide range of diseases in an efficient way. Our models use different window lengths for the training period, calculate a robust query-specific lag, denoise the data with a wavelet transform and identify relevant queries. We apply our models to worldwide unmodeled diseases to evaluate the utility of internet-based infectious disease surveillance to forecast one and two week incidences of Australian notification data.

## Results

### Search Term Selection and Internet Search Metrics

The number of search terms identified using Google Correlate for each disease ranged from zero to 1799 (out of a potential 1800 terms; Google Correlate returns up to 100 results per search). Once the lists were processed to remove duplicates and irrelevant terms, the identified keywords were concatenated with keywords identified in our previous study^22^. The final lists of search terms ranged from 69 unique terms for pneumococcal disease through to two terms for Murray Valley Encephalitis and botulism. In total, 197 unique search terms were identified and search metrics for these were downloaded for the period 2009–13. Weekly data from Google Trends were available at national level for 106 search terms. Ultimately, the number of search metrics available for each disease ranged between a single term and 34 terms (Table 1).

### Descriptive data analysis

Spearman’s rank correlations for the 24 diseases analysed in this study are presented in Table 2. There were marked differences in the level of correlation exhibited between disease notification and identified search terms; at national level eight diseases exhibited a strong correlation (0.600–0.799). Such measure was used to prioritise disease for further analysis and only the top 12 ranked diseases in Table 2 were analysed with our predictive linear models.

### Model construction and performance

**Figure 1:**
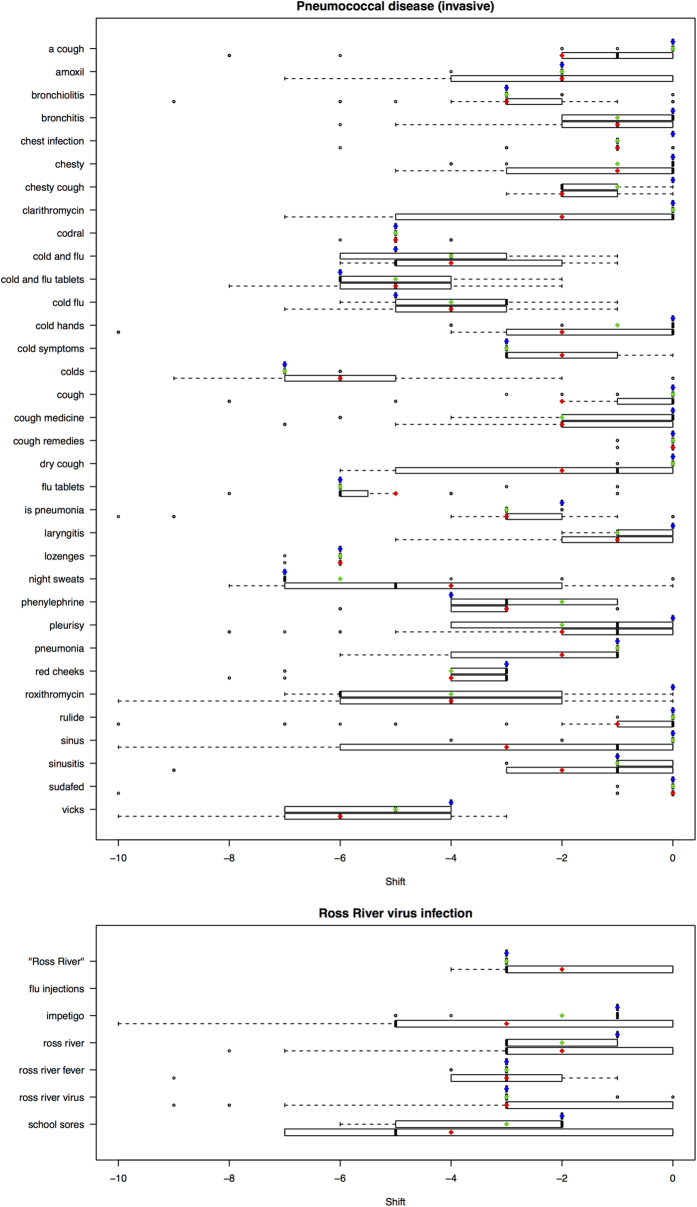
Boxplots of cross-correlation results for search terms and pneumococcal disease or Ross River virus infection. Cross-correlations were estimated using a shifting 52 or 104-week window over a 156 week (2009–11) period or for the entirety of the 156-week period. Red, green and blue dots indicate the mean best cross correlation for the 52, 104 and 156-week period respectively; dark lines indicate the median.

A total of 144 statistical linear models were fitted and tasked with producing one and two week predictions of disease notifications; 12 models were built for each of the 12 top ranked diseases. Prediction accuracy was assessed based on the Mean Square Error of Prediction (MSEP). Model performance varied markedly both between models and between diseases (Table 3). In total, models with MSEP lower than 0.40 were observed for two diseases: Pneumococcal disease and Ross River virus infection. We observed that the two highest ranked diseases by Spearman correlation (Table 2), Gonococcal Infection and Varicella zoster (Shingles), were among the worst predicted diseases (Table 3). This clearly shows that a query like “discharge” can be strongly correlated to the Gonococcal disease over a long period of time (2009–2013), but not predictive enough of this disease over 2012 and 2013. Cross-correlation results for the top two performing diseases are presented in Fig. 1. For these two diseases the most robust correlation for the 52- and 104-week shifting windows was commonly obtained for a greater advance of the search metrics over the notifications than for the single 156-week cross-correlation.

The performance of a model was assessed on both one-week and two-week estimates. The best performing model for one-week estimates was the 104RC model for pneumococcal disease (MSEP = 0.278), followed by the 156WC Ross River virus infection model (MSEP = 0.288). The results were largely similar for the two-week estimates (Table 3); however, the 156RC (MSEP = 0.293) model for Ross River virus infection exhibited a higher prediction accuracy than the 156WC model (MSEP = 0.303). Wavelet transformation to denoise data improved estimate accuracy in 43% (6/14) of the best performing models for one week estimates and 50% (7/14) for the two-week estimates. Finally, the training period for the best performing models differed between diseases (of the best performing models, 14 were obtained with 52 weeks of data, 7 with 104 weeks and 7 with 156 weeks), but was largely consistent within a disease.

**Figure 2:**
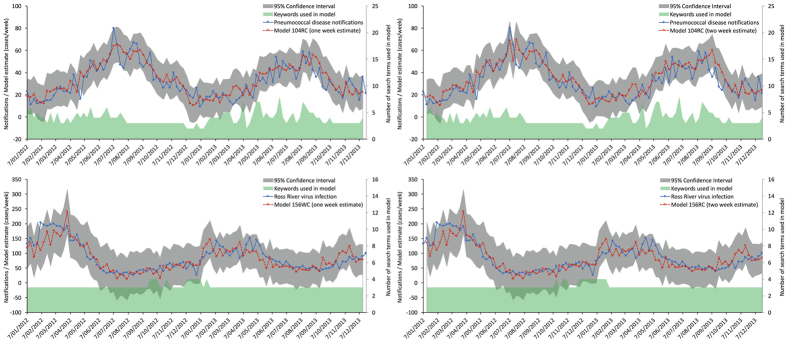
One (left) and two (right) week models for pneumococcal disease (top) and Ross River virus infection (bottom). Solid blue line indicates notifications; broken red line indicates the model estimate; grey shading indicates the 95% confidence interval; and the green shading at the bottom indicates the number of keywords used in the model to create the estimate.

Disease notifications, one week and two-week model estimates for the best performing pneumococcal disease and Ross River virus infection models are shown in Fig. 2. The number of search terms used to build the model for the corresponding time points are also displayed. Over the course of the validation period, 22 of the 34 search terms related to peumococcal disease were used at least once in the 104RC model; nine terms were used at least ten times (supplementary material). The number of search terms used for the estimates ranged from two to eight (mean 3.88; standard deviation 1.21). For Ross River virus infection, four of the six terms with data were used at least once in the 156WC and 156RC models and three terms were used for every prediction. The fourth term was selected in less than 1.2% of the models.

Finally, models were built for Ross River virus infection and pneumococcal disease using state level data. Owing to the loss of resolution in Google Trends data when focusing on smaller geographical areas, models could only be produced for New South Wales, Queensland and Victoria (see supplementary material). Both resolution and number of search metrics available for construction of state models were attenuated, affecting performance compared to national models.

## Discussion

The majority of previous studies into internet-based surveillance systems have predominantly focused on influenza^6^,^23^ and many made use of specific aspects of the disease such as seasonality. This study aimed to investigate the capacity of internet-based approaches to help monitoring a wide range of seasonal and non-seasonal infectious diseases in Australia. The results were consistent with our previous study that assessed the level of correlation between monthly search metrics and disease notification^14^. Based on the Spearman correlation results, eight diseases exhibited high degree of correlation with the investigated search metrics and were identified as having promise for nowcasting. Some studies have reported the use of internet metrics for monitoring some of these diseases^13^,^17^,^24^,^25^,^26^,^27^. It is, however, difficult to provide a meaningful, direct comparison between these studies and ours owing to the different methodologies used, search engines targeted, geographical regions analysed and types of notification data used. For instance, our attempt at modelling influenza led to lower nowcasting accuracy compared to the literature and that could be explained by a different behaviour of Google users in Australia vs USA or UK. Nonetheless, the results of this study support our previous assertion that internet-based surveillance systems have a wider potential application than is currently recognised and that internet-based surveillance systems appear to show best promise for monitoring vector-borne and vaccine-preventable diseases^14^.

Predictive linear models were fitted for the top performing 12 diseases, as ranked by Spearman correlation coefficients (Table 2). Functional linear models that provided accurate nowcasting of up to two weeks ahead of Australian notification data were created for two diseases of international interest: pneumococcal disease and Ross River virus infection (Fig. 2). Invasive pneumococcal disease is a vaccine-preventable disease of significant concern worldwide. The burden of disease for invasive pneumococcal disease, however, disproportionately affects infants and elderly people. Yearly incidence rates in Australia range between 6.7 and 12.3 notifications per 100,000 people^28^ and case fatality rates for persons under 5 and over 65 years of age are reported at upwards of 1.5% and 13.2%, respectively^29^. Ross River virus infection is the most widely spread arthropod-borne disease in Australia, reportedly accounting for around two-thirds of all notifications for mosquito-borne diseases^30^. Ross River virus is endemic to Australia and over the period modelled (2009–2013), 24,612 notifications were made; this equated to an incidence of between 18.6 and 23.3 cases per 100,000 persons per year. Ross River virus infection is of particular concern in Australia and regionally, owing to its potential to cause large outbreaks and to modelling that suggests land practices and climate change are likely to extend vector range and activity^31^. Whilst it would be remiss to suggest that the models that are presented in this publication are ready for deployment, our work clearly demonstrates a framework on which to produce actionable predictive models for both Ross River virus infection and invasive pneumococcal disease in Australia.

Twelve different predictive modelling approaches (Table 4) were applied to each of the top performing 12 diseases to predict one and two-week nowcasts of disease incidence. Different strategies were applied that increased accuracy and robustness of modelling. These include choice of model training period lengths, calculation of robust keyword-specific lag values, wavelet transformations of raw data and a continuous keyword selection. The classical linear model approach that serve as a reference was 156RS (Table 4; 156 weeks training period, raw data and set selection of keywords); this model was always outperformed by our alternative modelling (Table 3). All twelve approaches were based on sparse linear models and identified robust keywords with the method “mht”^32^. Mht models directly include multiple hypotheses testing using random subsamplings to account for the low numbers of time points while identifying the most relevant keywords to each disease. Selection of keywords is of the utmost importance to improve the accuracy of linear models because search metrics are influenced by human behaviour (such as health information seeking behaviour by the surveilled population)^6^,^33^,^34^; driven by media^33^,^35^,^36^,^37^ or fear^38^; are reliant upon technology and as such can be heavily influenced by this; and are reliant upon internet access.

To refine the keyword selection approach, our study considered to fit a new model with keyword selection for each time point (continuous selection). Such modelling strategy enables models to account for shifts, whether subtle or marked and does not assume seasonality. In addition, our systematic approach that uses the mht method does not require manual selection of parameters^32^ for the robust query selection process and was shown to detect switches in search behaviour that may affect model performance and adjust accordingly.

For the two diseases with best results (pneumococcal disease and Ross River virus infection), the model that employed a continuous keyword selection method outperformed its direct, set counterpart in 17 instances compared to 6 (Table 3; 52RC vs 52RS; 52WC vs 52WS; etc). These results suggest that approaches to modelling that allow more frequent updating of model parameters may be more suitable to internet-based data. We did not however apply those models for challenging periods such as pandemics.

Our study investigated the potential benefit of using wavelet transformation of internet search metrics data as a low pass filter prior to statistical modelling. We hypothesised that smoothing raw data by removing high frequency noise might enhance the link between a keyword and a disease. Wavelets have a number of applications^39^,^40^,^41^,^42^; they have not previously been applied to Internet search metrics for use in health surveillance. While wavelets did improve predictive performance for some diseases, the level of improvement was minimal and was somewhat inconsistent. We found that wavelet transform was beneficial in specific cases (e.g. Ross River virus infection, Chlamydial infection) but only marginally improved MSEP results by up to 5% compared to the raw data. Such results highlight the high diversity of patterns in internet data.

There has tended to be a philosophy that “more is better” with regard to data input into model construction. As is discussed above, search metrics are highly dynamic and we hypothesised that the use of time series that are too long may actually reduce both the accuracy and robustness of models as longer time series may mask emerging trends. We fitted models using training periods of one, two and three years to assess this (Table 3); the best performing models for each of the twelve diseases modelled in this study tended to favour one or two years data against three years. Therefore, the use of shorter training periods for models appeared to be better suited for short to medium term shifts in search behaviours. A future avenue to consider to further improve robustness of internet-based surveillance approaches would be to develop models that give greater weighting to more recent data.

This study showed that calculating lags from large data sets may in fact hide variability. Our proposed approach that determines the most “robust” value helps fitting stable models. In fact, the predictive ability of models may extend beyond 2 weeks depending on the best lag value identified.

The study presented interesting and promising outcomes, but also highlighted some shortcomings related to data availability at the present time of the analyses. First and foremost, the datasets available to us were relatively small, covering only 5 years; this was a function of the quality of Google Trends data prior to 2009^14^. We acknowledge that fitting models over longer periods could account for changes in community behaviour and provide some other interesting and valuable information regarding not only model performance, but also shifts in community behaviour and possibly health related knowledge. It is possible that the inclusion of particular search term into a model may be indicative of shifts in community knowledge or feeling towards a particular risk/treatment/preventative measure for a monitored disease. Secondly, this study looked at a very small subset of 197 unique search terms, compared to 50 million terms for the original Google Flu Trends model. Data access to the broad scientific community is restricted by Google, with the exception of non-standardised data available to selected research groups (https://www.google.org/flutrends/about/). However, while manual sorting of search metrics was a necessity for this study, our statistical models could easily handle several thousand search terms to create more robust predictive systems. Thirdly, we used *standardised* time series from Google Trends (see Results), which affects the statistical analysis. Indeed, a new time point added to an existing time series may alter the standardisation of the whole time series and thus impact on the modelling. Finally, the performance of our models may have been affected by noise introduced by search metrics of more than one word that Google Trends can aggregate with several similar search terms.

The use of internet search metrics for tracking and predicting infectious diseases is currently an area of significant interest. This study adds to the existing body of knowledge in a number of areas. On the one hand, this publication presents, to our knowledge, the first search metric based surveillance system in Australia for two diseases of international concern: Ross River virus and invasive pneumococcal disease. On the other hand, this study explored four extensions of classical linear models, based on varying training period length, lag calculation, wavelet data transformation and robust keyword selection. All four approaches exhibit strong promise for specific diseases, paving the way to novel systems able to accommodate for the dynamic nature of internet-based data to generate actionable infectious disease surveillance systems that are both accurate and robust.

## Methods

### Notations

We denote by n = 260 the total number of time points, where each time point corresponds to a week of aggregated data, ranging from 2009-01-10 to 2013-12-28. For a specific disease, let p be the number of associated search metrics. We denote by ***Y*** a vector of length n containing the notifications and by ***X*** a ***n*** × ***p*** matrix of p concatenated search metrics, where ***X***^***j***^, ***j*** = 1, …, ***p*** contains the occurrence of a specific search metric over all time points. In addition, we denote by 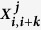 the occurrence of the search metric j between the time points i and i + k.

### Infectious Disease Surveillance Data

Surveillance data on notifiable infectious diseases were provided by Australia Government Department of Health (DoH) from the National Notifiable Disease Surveillance System (NNDSS)^28^. Weekly notifications (case numbers) aggregated at state/territory and national level, were provided for the years 2004 through 2013, inclusive. The Australian government monitors sixty-four diseases through the NNDSS; a full list of notifiable diseases in Australia and case definitions can be accessed through the DoH webpage^43^. For this study, analyses were restricted to the 24 diseases identified in our previous publication as having the most potential for use in digital surveillance systems^14^. These were: Barmah Forest virus infection, botulism, chikungunya virus infection, chlamydial infection, cryptosporidiosis, dengue virus infection, gonococcal infection, hepatitis A, hepatitis B (newly acquired), hepatitis B (unspecified), hepatitis C (unspecified), influenza (laboratory confirmed), legionellosis, leptospirosis, listeriosis, measles, meningococcal disease (invasive), Murray Valley encephalitis virus infection, pertussis, pneumococcal disease (invasive), Ross River virus infection, varicella zoster (chickenpox), varicella zoster (shingles) and varicella zoster (unspecified).

### Search Term Selection and Scraping of Internet Search Trend Data

A similar approach to search term selection was employed as has previously been described^14^. Briefly, two approaches were employed. Firstly, terms related to diseases, aetiological agents and colloquialisms were manually identified. Secondly, Google Correlate (www.google.com/trends/correlate) was queried using weekly surveillance data (described above) to identify the search terms with the highest degree of correlation at state and national level for the periods 2006–13 and 2009–13. Using this approach, up to 1800 search terms were downloaded from Google Correlate for each of the 24 diseases. These were manually sorted; any term related to the queried notifiable disease was included, regardless of the nature of the potential association and combined with manually identified search terms (see supplementary material for full list of terms).

Search frequencies for the terms of interest were collected from Google Trends (www.google.com/trends/) using a custom script (see supplementary material). All data was downloaded at state/territory and national levels (for Australia) for the period January 2009 to July 2014. Data collection were only performed back to 2009 as our previous work indicated data quality prior to 2009 to be insufficient^14^. All data extractions were performed on the 1^st^ of September, 2014. Google Trends provides data as a standardised time series (the data point with the highest search frequency is given a value of 100 and all other points scaled accordingly). The level of temporal aggregation (weekly or monthly) is determined by the period analysed and the search frequency; this cannot be specified by the user. Any data not returned as a weekly time series were discarded. These standardised time series collected from Google Trends will be referred to as “search metrics” henceforth.

### Descriptive data analysis

To identify correlated time-series and prioritise diseases for further investigation, correlation analyses based on Spearman’s rank correlation were performed between disease notification data and search metrics on 2009–2013. Spearman’s rank correlation was chosen over Pearson correlation so as to prioritise monotonic relationship. Correlations were performed at both state-level and national-level; each data set analysed contained 260 data points.

### Model construction and validation

All models produced in this study were built using data from the 2009–2011 seasons (inclusive, 156 weeks); the 2012 and 2013 season data were reserved for model validation (104 weeks). For each disease, 12 linear models were fitted; models differed in the length of the modelling window (52, 104 or 156 weeks), the data used (raw search metric data as extracted from Google Trends or data that had been denoised using DaubLeAsymm family of wavelets, as described in supplementary material), and on the selection process for keywords (continuous or set, see details below). Model characteristics are summarised in Table 4. Based on prior observations^13^, we assumed a two-week lag in the reporting process of disease notifications, but not in the search metrics. Consequently, models in this study were tasked with producing one and two week predictions of disease notifications. See supplementary material for details on the predictive models.

### Time-series cross-correlations to evaluate best lag for each search metrics

Firstly, investigation of the nature of the association between the search metrics and disease notification data was undertaken by performing time-series cross-correlations^44^ of the 2009–2011 data using the statistical R programming language^45^. Lag values for search metrics data ranging from −10 to 0 were calculated. As previously discussed this range allowed assessment of biologically plausible associations, relevant to the development of early warning systems^14^. Briefly, for a time series of k time points (52, 104 or 156), a correlation is calculated between a vector of notifications that has been shifted for a given lag 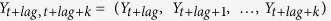 and a search metric 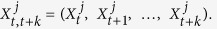
Negative lag values were of most interest within the context of this study as they indicated that the search metrics lead notification data, which allow prediction of the notifications up to 10 weeks in advance. The aim of the cross-correlation analysis was to identify the best shift to apply to each search metrics for subsequent analysis. Contrary to the traditional approach that calculate cross-correlations across the entire 156-week period (2009–2011), our approaches estimated a series of cross-correlations using a shifting 52 or 104-week window; these periods were chosen to address potential season effects that may influence results. Regarding the 52-week window, a cross-correlation analysis was performed using only 52 week’s data (*k* = 52), starting week 10 in 2009 (weeks 10 to 62, *t* = 10). The correlation was recorded for each lag (lag = −10, …, 0). The window was then moved forward by one week (to encompass weeks 11 to 63, *t* = 11) and the process was repeated until the entire 156-week data set (2009–2011) was analysed. Using these results over 95 weeks (*t* = 10 to *t* = 104), an average correlation for each lag value was calculated. This approach enabled to assess the robustness of each lag value for each search metric/disease. The best lag value with the highest averaged correlation was identified by this process as being the most robust value and was defined as the value 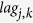 that maximises


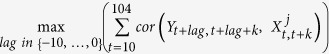


for a specific search metric *j* and a specific length of the shifting window *k*, where *cor* is the correlation. An identical approach was used using a shifting 104-week window (*k* = 104, *t* = 10 to *t* = 52). Each search term was shifted by the best lag value and the adjusted time-series were used in the subsequent construction of models. Let denote 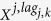 the adjusted time-series for search metric *j* based on the shifting window of length *k*, with 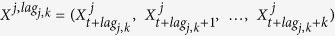. 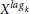 is the *n* × *p* matrix of the *p* adjusted time-series of search metrics for a shifting window of length *k* and is defined as 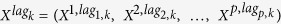. Note that for *k* = 156, there is no average lag since all the training data was used for the 156-week window.

### Continuous or set search metrics selection in linear models

The relationship between a disease notification and search metric data was assumed to be linear, modelled by


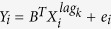


where *Y*_*i*_ is a single notification at time *i*, 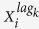, is a vector that contains all search metrics corresponding to time *i* and shifting window of length *k* (after being adjusted by their respective best lag), *B* is an unknown parameter to be estimated and *e_i_* is independent zero-centered noise. Since most queries should be unrelated to the notification, most entries of *B* are zero (sparse matrix). To perform keyword selection, we considered the mht procedure as it was shown to outperform common variable selection statistical methods in high-dimensional linear models where the number of observations is smaller than the number of parameters^32^. mht relies on multiple random subsamplings to account for the low number of observations (52, 104 or 156) and to retain the most relevant queries only in the resulting linear model. Relevant queries are determined as the most stable across multiple random subsamplings in the mht procedure. Keywords used in models were either selected based on the whole 2009–2011 training period with 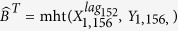 that was input in every subsequent models as a set selection with, 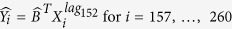, or, alternatively, as continuous selection were reselected each week with, 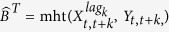, depending on the preceding 52, 104 or 156 weeks that was then input to predict the next two weeks of disease notifications with 
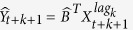
 and 
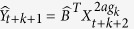
.

### Evaluation of performances

Mean Square Error of Prediction (MSEP) were calculated for each disease to evaluate the performance of each of the twelve sparse linear models across all 2012–2013 seasons (104 data points in total) as defined by:


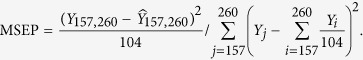


using the notations described earlier.

### Ethics

Ethics clearance for this project was approved by The University of Queensland Medical Research Ethics Committee (approval number 2013000413) and Queensland University of Technology Medical Research Ethics Committee (approval number 1400000721).

## Additional Information

**How to cite this article**: Rohart, F. *et al*. Disease surveillance based on Internet-based linear models: an Australian case study of previously unmodeled infection diseases. *Sci. Rep.* **6**, 38522; doi: 10.1038/srep38522 (2016).

**Publisher's note:** Springer Nature remains neutral with regard to jurisdictional claims in published maps and institutional affiliations.

## Supplementary Material

Supplementary Information

## Figures and Tables

**Table 1:** Summary of the number of search terms identified and used in this study for each disease.

*Disease*	*Terms identified*	*Unique search terms*	*Relevant terms*	*Previously identified*	*Final list*	*Terms with data*
Pneumococcal disease (invasive)	777	304	54	35	69	34
Varicella zoster (Chickenpox)	115	115	9	8	15	13
Varicella zoster (Shingles)	953	710	6	8	14	11
Influenza (laboratory confirmed)	1799	701	16	14	20	8
Varicella zoster (unspecified)	637	532	2	8	9	7
Gonococcal infection	909	663	2	6	8	6
Ross River virus infection	1316	931	19	6	19	6
Barmah Forest virus infection	420	271	11	2	12	6
Dengue virus infection	803	505	8	6	11	5
Hepatitis B (unspecified)	24	24	0	5	5	5
Hepatitis A	644	428	2	4	6	5
Hepatitis B (newly acquired)	0	0	0	5	5	5
Pertussis	1629	1287	5	5	6	4
Hepatitis C (unspecified)	144	120	0	4	4	4
Meningococcal disease (invasive)	0	0	0	9	9	4
Chlamydial infection	1261	431	2	2	3	3
Leptospirosis	162	130	3	6	8	3
Murray Valley encephalitis virus infection	913	538	0	2	2	2
Cryptosporidiosis	795	424	2	2	4	2
Chikungunya virus infection	414	253	0	2	2	2
Listeriosis	216	127	2	2	4	2
Measles	795	556	1	2	3	1
Botulism	464	347	0	2	2	1
Legionellosis	0	0	0	3	3	1

**Table 2:** Spearman’s rho correlation coefficients for diseases notifications-search metrics for the period 2009–13.

Disease	Top ranked search term	ACT	NSW	NT	QLD	SA	TAS	VIC	WA	AUS	p-value
Gonococcal infection	discharge		0.795	−0.028	0.298	0.205		0.556	0.265	0.786	<0.00001
Varicella zoster (Shingles)	diarrhea				−0.400	0.546		0.578	0.633	0.762	<0.00001
Pneumococcal disease (invasive)	bronchitis		0.558		0.338			0.402		0.753	<0.00001
Ross River virus infection	"ross river"		0.456		0.082					0.742	<0.00001
Pertussis	whooping		0.661		0.489			0.525	0.700	0.651	<0.00001
Chlamydial infection	blood test		0.540	0.038	0.208	0.433		0.390	0.557	0.634	<0.00001
Varicella zoster (unspecified)	blood test			−0.023	0.297	−0.404		0.316	0.400	0.628	<0.00001
Varicella zoster (Chickenpox)	conjunctivitis				0.071			0.380		0.624	<0.00001
Cryptosporidiosis	ross river virus									0.569	<0.00001
Barmah Forest virus infection	ross river virus									0.539	<0.00001
Dengue virus infection	dengue	−0.036	0.337		0.508			0.569		0.507	<0.00001
Influenza (laboratory confirmed)	flu symptoms	0.344	0.290		0.589			0.485		0.423	<0.00001
Leptospirosis	ross river		0.110		0.059					0.405	<0.00001
Measles	measles		0.198		0.263			0.119		0.367	<0.00001
Hepatitis C (unspecified)	hepatitis		0.193		0.116	−0.149		0.142	−0.035	0.297	<0.00001
Hepatitis A	hepatitis a		−0.167							0.293	<0.00001
Murray Valley encephalitis virus infection	murray valley encephalitis									0.265	<0.0001
Legionellosis	legionnaires					0.108				0.237	<0.0001
Hepatitis B	hepatitis		0.183		0.097	−0.060		0.067	−0.007	0.230	<0.001
Meningococcal disease (invasive)	rulide									0.222	<0.001
Chikungunya virus infection	dengue		−0.023		0.104			0.140		0.194	0.0013
Hepatitis B (newly acquired)	hepatitis		−0.116		−0.014	−0.064		0.157	−0.037	0.123	0.0333
Listeriosis	listeria	−0.010								0.091	0.0906
Botulism	botulism									0.053	0.2299

The table only contains the search term with the highest degree of correlation for each disease; see supplementary material for a full list of search terms and correlation coefficients. p-values relate to correlations at national level. Empty cells indicate that Google Trends data were not available.

**Table 3:** Model performance for 1 week (top) and 2 week estimates (bottom), as assessed by Mean Square Error of Prediction.

1 Week estimate												
	*52RC*	*52WC*	*52RS*	*52WS*	*104RC*	*104WC*	*104RS*	*104WS*	*156RC*	*156WC*	*156RS*	*156WS*
Gonococcal infection	1.183	1.237	1.212	1.229	**1.181**	1.301	1.274	1.368	1.268	1.340	1.356	1.358
Varicella zoster (Shingles)	0.970	1.105	**0.840**	0.850	0.907	0.964	0.946	0.937	0.954	1.008	0.967	0.976
Pneumococcal disease (invasive)	0.478	0.510	0.523	0.548	**0.278**	0.347	0.564	0.376	0.420	0.396	0.435	0.437
Ross River virus infection	0.394	0.542	0.465	0.514	0.365	0.537	0.351	0.478	0.289	**0.288**	0.290	**0.288**
Pertussis	1.418	**1.394**	1.447	1.416	2.053	1.659	2.206	2.118	2.299	2.265	2.240	2.189
Chlamydial infection	1.089	1.019	0.983	0.968	0.832	**0.789**	0.832	**0.789**	0.847	0.826	0.847	0.826
Varicella zoster (unspecified)	0.771	**0.764**	0.830	0.845	0.892	0.942	0.883	0.909	0.994	0.957	1.014	1.014
Varicella zoster (Chickenpox)	0.862	0.879	**0.746**	0.758	0.773	0.805	0.760	0.760	0.875	0.926	0.884	0.861
Cryptosporidiosis			**1.031**	1.050			1.071	1.089			1.048	1.049
Barmah Forest virus infection	1.527	1.520	**1.072**	1.249	1.188	1.214	1.201	1.267	1.180	1.189	1.197	1.203
Dengue virus infection	1.362	1.748	1.355	1.417	1.220	1.263	1.149	1.177	**1.101**	1.110	1.334	1.342
Influenza (laboratory confirmed)	0.425	0.463	**0.406**	0.438	0.461	0.485	0.461	0.490	0.718	0.681	0.915	0.922
**2 Week estimate**												
Gonococcal infection	1.213	**1.145**	1.240	1.217	1.231	1.235	1.306	1.181	1.317	1.266	1.367	1.267
Varicella zoster (Shingles)	1.015	1.071	0.846	**0.820**	0.903	0.907	0.950	0.918	0.918	1.020	0.979	0.966
Pneumococcal disease (invasive)	0.388	0.496	0.529	0.473	**0.311**	0.374	0.577	0.454	0.403	0.462	0.446	0.495
Ross River virus infection	0.425	0.449	0.439	0.452	0.364	0.465	0.350	0.378	**0.293**	0.303	0.295	0.304
Pertussis	1.575	**1.562**	1.594	1.571	2.166	1.922	2.399	2.313	2.453	2.436	2.397	2.378
Chlamydial infection	1.052	1.103	0.992	1.060	**0.856**	0.991	**0.856**	0.991	0.884	0.948	0.884	0.948
Varicella zoster (unspecified)	0.886	**0.813**	0.869	0.878	0.951	0.854	0.929	0.854	1.034	0.980	1.046	0.995
Varicella zoster (Chickenpox)	0.862	0.771	**0.768**	0.789	0.832	0.930	0.769	0.781	0.969	1.060	0.923	0.908
Cryptosporidiosis			1.092	1.098			1.089	1.096			**1.058**	**1.058**
Barmah Forest virus infection	1.643	1.677	**1.129**	1.257	1.202	1.265	1.221	1.249	1.204	1.200	1.221	1.222
Dengue virus infection	1.373	1.527	1.335	1.344	1.269	1.277	1.178	1.175	1.172	**1.147**	1.341	1.309
Influenza (laboratory confirmed)	0.473	0.464	0.428	**0.420**	0.490	0.473	0.501	0.481	0.769	0.734	0.966	0.966

The highest performing models for each disease are indicated in bold. Model characteristics are described in Table 4.

**Table 4:** Summary of model characteristics.

Model	Training period^1^	Google Trends data^2^	Keyword selection^3^	Model Name^4^
1	52 weeks	Raw data	Continuous	52RC
2	52 weeks	Wavelet transformed	Continuous	52WC
3	104 weeks	Raw data	Continuous	104RC
4	104 weeks	Wavelet transformed	Continuous	104WC
5	156 weeks	Raw data	Continuous	156RC
6	156 weeks	Wavelet transformed	Continuous	156WC
7	52 weeks	Raw data	Set	52RS
8	52 weeks	Wavelet transformed	Set	52WS
9	104 weeks	Raw data	Set	104RS
10	104 weeks	Wavelet transformed	Set	104WS
11	156 weeks	Raw data	Set	156RS
12	156 weeks	Wavelet transformed	Set	156WS

^1^The training period denotes how many weeks data are available to the model for fitting, keyword selection and wavelet construction. This period was also used to determine the best lag for keywords used in these models (but was restricted to the 2009–2011 data).

^2^Indicates the search metrics data available for the model.

^3^In producing forecasts for holdout data (2012–2013), continuous models are able to reselect keywords at each time point using the previous 52, 104 or 156 weeks data; set models use a selection of keywords determined using only the 2009–2011 data.

^4^Models are named using a combination of the number of weeks data visible to them (52/104/156), format of search metric data (raw/wavelet transformed; R/W) and the method of keyword selection (continuous/set; C/S).
