# Supplementary material for: Disease surveillance based on Internet-based linear models: an Australian case study of previously unmodeled infection diseases
Source: Sci Rep. 2016 Dec 20;6:38522. doi: 10.1038/srep38522 (PMC5172376; doi:10.1038/srep38522)
Supplement: Supplementary Information [file srep38522-s1.pdf]

Disease surveillance based on internet-based linear models: an Australian case study of previously unmodeled infection diseases

Florian Rohart[+]*, Gabriel J. Milinovich[+], Simon M. R. Avril, Kim-Anh Lê Cao, Shilu Tong and Wenbiao Hu

[+] These authors contributed equally to this work

*Author for correspondence

Authors affiliations:
Florian Rohart PhD
The University of Queensland
St Lucia, 4072
QLD, Australia
f.rohart@uq.edu.au

Gabriel J. Milinovich PhD
School of Medicine
The University of Queensland
St Lucia, 4072
QLD, Australia

School of Public Health and Social Work
Queensland University of Technology
Kelvin Grove, 4056
QLD, Australia
g.milinovich@uq.edu.au
+61 404 153 413

Simon M. R. Avril M.Eng (Software)
Freelance developer
simon.m.r.avril@gmail.com

Kim-Anh Lê Cao PhD
The University of Queensland
Woolloongabba, 4102
QLD, Australia
k.lecao@uq.edu.au

Professor Shilu Tong PhD
School of Public Health and Social Work
Queensland University of Technology
Kelvin Grove, 4056
QLD, Australia

s.tong@qut.edu.au

Wenbiao Hu PhD
Queensland University of Technology
Kelvin Grove, 4056
QLD, Australia
w2.hu@qut.edu.au

Full list of Google Trends terms analysed

The following section lists all search terms analysed in this study and provides tables for each disease indicating the correlation (Spearman rho) between disease notification data and search metrics for the period 2009-14. Blank cells indicate that there was insufficient data to determine the degree of correlation.

Unique Terms*:* "Barmah forest"; "Chicken pox"; "hep a"; "hep b"; "hep c"; "Ross River"; a cough; alphamox; amoxil; barmah forest; barmah forest virus; biaxsig; blood in mucus; blood in phlegm; blood test; botulism; bromhexine; bronchiolitis; bronchitis; bronchitis contagious; bruise; bruise easily; bruised; cairns flood; ceclor; cervical mucus; chest infection; chest infections; chesty; chesty cough; Chikungunya; chilblains; Chlamydia; cilamox; clarithromycin; codeine linctus; codral; codral cold and flu; cold and flu; cold and flu tablets; cold flu; cold hands; cold hands and feet; cold symptoms; colds; conjunctivitis; conjunctivitis contagious; cough; cough medicine; cough remedies; cough remedy; cough suppressant; coughs; cramping; cramps; Cryptosporidiosis; Cryptosporidium; cymbalta; decongestant; demazin; Dengue; dengue fever; dengue fever symptoms; dengue mosquito; dengue symptoms; dextromethorphan; dextromethorphan hydrobromide; diarrhea; difflam; dimetapp; discharge; dry cough; duro tuss; expectorant; flash floods; flood; flood australia; flood damage; flood damaged cars; flood recovery; Flu; flu and pregnancy; flu contagious; flu cough; flu incubation; flu incubation period; flu injection; flu injections; flu pregnancy; flu shots; flu symptoms; flu tablets; flu treatment; flu vaccinations; fluvax; foot and; foot and mouth; foot and mouth disease; gonococcal; gonorrhea; gonorrhoea; h1n1 flu; h1n1 vaccine; hand foot; hand foot and mouth; hep; hepatitis; Hepatitis A; Hepatitis B; Hepatitis C; hives rash; hooping; human temperature; impetigo; Influenza; influenza a; influenza a virus; influenza symptoms; influenza vaccine; influvac; is pneumonia; is pneumonia contagious; kalixocin; laryngitis; legionella; Legionellosis; legionnaires; lemsip; leptospirosis; linctus; listeria; listeriosis; lozenges; measles; measles outbreak; meningitis; meningococcal; mouth disease; Murray Valley; Murray Valley encephalitis; myxomatosis; nasal congestion; neomycin; neuritis; night sweats; optic neuritis; panvax; pathology; Pertussis; phenomia; phenylephrine; phenylephrine hydrochloride; pholcodine; pleurisy; pneumococcal; pneumonia; pristiq; red cheeks; ;rheumatology; ;rikodeine; ;robitussin; ;ross river; ;ross river fever; ;ross river fever symptoms; ross river virus; ross river virus symptoms; roxar; roxithromycin; rulide; school sores; senega; senega and ammonia; shingles; sinus; sinusitis; staph infection; strepsils; sudafed; swine flu symptoms; symptom checker; symptoms of

dengue; symptoms of dengue fever; symptoms of pneumonia; symptoms of swine; symptoms of swine flu; tamiflu; tamiflu side effects; townsville flood; Varicella; vicks; water flood; white creamy discharge; white discharge; whooping; whooping cough; whooping cough in adults; whooping cough treatment

Barmah Forest virus infection: "Barmah forest"; barmah forest; barmah forest virus; flood australia; flood damage; flood damaged cars; flood recovery; myxomatosis; ross river; ross river fever; ross river virus; water flood

Term	ACT	NSW	NT	Qld	SA	Tas	Vic	WA	Aus
ross river virus									0.539
ross river		0.358		0.251					0.414
ross river fever									0.399
flood australia	-0.016				0.194				0.250
water flood					0.112				0.237
flood damaged cars									0.082
"barmah forest"									
barmah forest									
barmah forest virus									
flood damage				0.182					
flood recovery		0.096		0.204					
myxomatosis									

Botulism: botulism; hives rash

Term	ACT	NSW	NT	Qld	SA	Tas	Vic	WA	Aus
botulism									0.053
hives rash									

Chikungunya virus infection: Chikungunya; Dengue

Term	ACT	NSW	NT	Qld	SA	Tas	Vic	WA	Aus
dengue		-0.023		0.104			0.140		0.194
chikungunya				-0.033			0.016	0.056	0.167

Chlamydial infection: blood test; Chlamydia; pathology

Term	ACT	NSW	NT	Qld	SA	Tas	Vic	WA	Aus
blood test		0.540	0.038	0.208	0.433		0.390	0.557	0.634
pathology	0.163	0.484		0.253	0.129		0.181	-0.008	0.572
chlamydia		0.458		0.213			0.295	0.493	0.516

Cryptosporidiosis: Cryptosporidiosis; Cryptosporidium; dengue mosquito; ross river virus

Term	ACT	NSW	NT	Qld	SA	Tas	Vic	WA	Aus
ross river virus									0.569
cryptosporidiosis									0.147
cryptosporidium							0.213		
dengue mosquito									

Dengue virus infection: bruise; bruise easily; bruised; Dengue; dengue fever; dengue fever symptoms; dengue mosquito; dengue symptoms; symptom checker; symptoms of dengue; symptoms of dengue fever

Term	ACT	NSW	NT	Qld	SA	Tas	Vic	WA	Aus
dengue	-0.036	0.337		0.508			0.569		0.507
dengue fever	-0.036	0.477					0.562		0.499
bruise		0.289		0.260			0.441		0.441
bruised		0.285		0.161			0.495		0.401
symptom checker									0.355

bruise easily									
dengue fever symptoms									
dengue mosquito									
dengue symptoms								0.022	
symptoms of dengue									
symptoms of dengue fever									

Gonococcal infection: cervical mucus; discharge; gonococcal; gonorrhea; gonorrhoea; pristiq; staph infection; white discharge

Term	ACT	NSW	NT	Qld	SA	Tas	Vic	WA	Aus
discharge		0.795	-0.028	0.298	0.205		0.556	0.265	0.786
white discharge		0.742		0.228			0.643		0.762
pristiq		0.642		0.273			0.513		0.594
cervical mucus									0.591
staph infection		0.659					0.544		0.524
gonorrhea		0.477					0.437		0.422
gonococcal									
gonorrhoea									

Hepatitis A: "hep a"; h1n1 vaccine; hep; hepatitis; Hepatitis A; panvax

Term	ACT	NSW	NT	Qld	SA	Tas	Vic	WA	Aus
hepatitis a		-0.167							0.293
panvax									0.256
hepatitis		-0.021		-0.008	-0.191		0.196	-0.198	0.146
hep		-0.026		-0.092			-0.246		-0.191
"hep a"									-0.302
h1n1 vaccine		0.119							

Hepatitis B (newly acquired): "hep b"; hep; hepatitis; Hepatitis B; rheumatology

Term	ACT	NSW	NT	Qld	SA	Tas	Vic	WA	Aus
hepatitis		-0.116		-0.014	-0.064		0.157	-0.037	0.123
hepatitis b		-0.002					-0.263		0.034
"hep b"									0.029
rheumatology									-0.072
hep		-0.055		-0.041			-0.184		-0.089

Hepatitis B (unspecified): "hep b"; hep; hepatitis; Hepatitis B; rheumatology

Term	ACT	NSW	NT	Qld	SA	Tas	Vic	WA	Aus
hepatitis		0.183		0.097	-0.060		0.067	-0.007	0.230
hepatitis b		0.005					-0.008		0.166
hep		-0.023		-0.167			-0.014		0.117
"hep b"									0.105
rheumatology									-0.056

Hepatitis C (unspecified): Hepatitis C; hepatitis; hep; "hep c"

Term	ACT	NSW	NT	Qld	SA	Tas	Vic	WA	Aus
hepatitis		0.193		0.116	-0.149		0.142	-0.035	0.297
hepatitis c		0.064					-0.104		0.266
"hep c"		-0.152							0.189
hep		0.105		-0.052			-0.010		0.027

Influenza (laboratory confirmed: Flu; flu and pregnancy; flu contagious; flu cough; flu incubation; flu incubation period; flu pregnancy; flu symptoms; flu treatment; h1n1 flu; human temperature; Influenza; influenza a; influenza a virus; influenza symptoms; swine flu symptoms; symptoms of swine; symptoms of swine flu; tamiflu; tamiflu side effects

Term	ACT	NSW	NT	Qld	SA	Tas	Vic	WA	Aus
flu symptoms	0.344	0.290		0.589			0.485		0.423
influenza		0.364		0.615			0.287		0.383
flu incubation period		0.105							0.362
human temperature									0.321
flu		0.265	0.095	0.276	0.386		0.239	0.280	0.313
flu incubation		0.150							0.308
tamiflu side effects									0.212
swine flu symptoms	0.342	0.150			0.322		0.118	0.305	0.107
flu and pregnancy									
flu contagious									
flu cough									
flu pregnancy									
flu treatment									
h1n1 flu		0.260					0.151		
influenza a									
influenza a virus									
influenza symptoms				0.099			0.100		
symptoms of swine		0.322		0.227	0.079		0.267	0.134	
symptoms of swine flu		0.334		0.224			0.266		
tamiflu					-0.021			-0.016	

Legionellosis: legionella; Legionellosis; legionnaires

Term	ACT	NSW	NT	Qld	SA	Tas	Vic	WA	Aus
legionnaires					0.108				0.237

legionella									
Legionellosis									

Leptospirosis: barmah forest virus; cairns flood; flash floods; flood; flood damaged cars; leptospirosis; ross river; townsville flood

Term	ACT	NSW	NT	Qld	SA	Tas	Vic	WA	Aus
ross river		0.110		0.059					0.405
cairns flood		0.074		0.182					0.288
flood damaged cars									0.102
barmah forest virus									
flash floods		0.052		0.108			0.094		
flood		0.193	-0.014		0.014		0.123	0.078	
leptospirosis									
townsville flood		0.116		0.165					

Listeriosis: listeria; listeriosis; neuritis; optic neuritis

Term	ACT	NSW	NT	Qld	SA	Tas	Vic	WA	Aus
listeria	-0.010								0.091
neuritis									-0.075
listeriosis									
optic neuritis		-0.050					0.064		

Measles: dengue symptoms; measles; measles outbreak

Term	ACT	NSW	NT	Qld	SA	Tas	Vic	WA	Aus
measles		0.198		0.263			0.119		0.367
dengue symptoms								-0.028	

measles outbreak		0.059					0.050		

Meningococcal disease (invasive): alphamox; amoxil; biaxsig; ceclor; cilamox; meningitis; meningococcal; roxar; rulide

Term	ACT	NSW	NT	Qld	SA	Tas	Vic	WA	Aus
rulide									0.222
meningococcal		0.018							0.180
meningitis		-0.059		-0.011			-0.010		0.025
amoxil									-0.066
alphamox									
biaxsig									
ceclor									
cilamox									
roxar									

Murray Valley encephalitis virus infection: Murray Valley; Murray Valley encephalitis

Term	ACT	NSW	NT	Qld	SA	Tas	Vic	WA	Aus
murray valley encephalitis									0.265
murray valley								-0.012	0.069

Pertussis: hooping; Pertussis; whooping; whooping cough; whooping cough in adults; whooping cough treatment

Term	ACT	NSW	NT	Qld	SA	Tas	Vic	WA	Aus
whooping		0.661		0.489			0.525	0.700	0.651
whooping cough		0.695		0.443			0.503	0.679	0.649
whooping cough in									0.413

adults									
pertussis									0.391
hooping									
whooping cough treatment									

Pneumococcal disease (invasive): a cough; alphamox; amoxil; blood in mucus; blood in phlegm; bromhexine; bronchiolitis; bronchitis; bronchitis contagious; chest infection; chest infections; chesty; chesty cough; chilblains; clarithromycin; codeine linctus; codral; codral cold and flu; cold and flu; cold and flu tablets; cold flu; cold hands; cold hands and feet; cold symptoms; colds; cough; cough medicine; cough remedies; cough remedy; cough suppressant; coughs; decongestant; demazin; dextromethorphan; dextromethorphan hydrobromide; difflam; dimetapp; dry cough; duro tuss; expectorant; flu tablets; is pneumonia; is pneumonia contagious; laryngitis; lemsip; linctus; lozenges; nasal congestion; night sweats; phenomia; phenylephrine; phenylephrine hydrochloride; pholcodine; pleurisy; pneumococcal; pneumonia; red cheeks; rikodeine; robitussin; roxithromycin; rulide; senega; senega and ammonia; sinus; sinusitis; strepsils; sudafed; symptoms of pneumonia; vicks

Term	ACT	NSW	NT	Qld	SA	Tas	Vic	WA	Aus
bronchitis		0.558		0.338			0.402		0.753
pneumonia		0.611	0.043	0.479	0.186		0.578	0.371	0.747
cold flu		0.550		0.387			0.276		0.715
codral									0.663
colds		0.406					0.338		0.654
cold and flu		0.493					0.259		0.653
cough		0.497	0.060	0.479	0.176		0.541	0.384	0.652
pleurisy									0.644
cold symptoms		0.401					0.327		0.631
chest infection		0.389					0.268		0.628
sudafed									0.605
laryngitis		0.324							0.586

night sweats		0.402					0.168		0.568
vicks		0.336					0.320		0.557
sinus		0.413	0.043	0.356	0.104		0.357	0.201	0.555
chesty									0.552
bronchiolitis									0.542
dry cough		0.343					0.280		0.542
a cough		0.306		0.214			0.213		0.534
cold hands									0.530
rulide									0.525
roxithromycin									0.521
is pneumonia									0.492
phenylephrine									0.462
sinusitis		0.270					0.237		0.460
cold and flu tablets									0.448
cough medicine									0.443
flu tablets									0.397
chesty cough									0.396
cough remedies									0.391
amoxil									0.323
clarithromycin									0.306
red cheeks									0.292
lozenges									0.249
alphamox									
blood in mucus									
blood in phlegm									

bromhexine									
bronchitis contagious									
chest infections									
chilblains								0.062	
codeine linctus									
codral cold and flu									
cold hands and feet									
cough remedy									
cough suppressant									
coughs									
decongestant									
demazin									
dextromethorphan									
dextromethorphan hydrobromide									
difflam									
dimetapp									
duro tuss									
expectorant									
is pneumonia contagious									
lemsip									
linctus									
nasal congestion									
phenomia									
phenylephrine									

hydrochloride									
pholcodine									
pneumococcal									
rikodeine									
robitussin									
senega									
senega and ammonia									
strepsils									
symptoms of pneumonia									

Ross River virus infection: "Ross River"; barmah forest; barmah forest virus; flu injection; flu injections; flu shots; flu vaccinations; fluvax; impetigo; influenza vaccine; influvac; myxomatosis; neomycin; ross river; ross river fever; ross river fever symptoms; ross river virus; ross river virus symptoms; school sores

**Term**	**ACT**	**NSW**	**NT**	**Qld**	**SA**	**Tas**	**Vic**	**WA**	**Aus**
"ross river"		0.456		0.082					0.742
ross river		0.467		0.016					0.732
ross river fever									0.626
ross river virus									0.623
school sores				0.250					0.608
impetigo		0.211							0.452
barmah forest									
barmah forest virus									
flu injection									
flu injections									
flu shots		-0.014							

flu vaccinations		-0.014							
fluvax									
influenza vaccine									
influvac									
myxomatosis									
neomycin									
ross river fever symptoms									
ross river virus symptoms							0.106		

Varicella zoster (Chickenpox): "Chicken pox"; clarithromycin; conjunctivitis; conjunctivitis contagious; cymbalta; foot and; foot and mouth; foot and mouth disease; hand foot; hand foot and mouth; kalixocin; mouth disease; pristiq; shingles; Varicella

**Term**	**ACT**	**NSW**	**NT**	**Qld**	**SA**	**Tas**	**Vic**	**WA**	**Aus**
conjunctivitis				0.071			0.380		0.624
hand foot and mouth				0.021			0.369		0.433
foot and mouth				0.022			0.395		0.423
hand foot				-0.026			0.330		0.420
clarithromycin									0.400
foot and			0.085	0.013	0.009		0.326	0.363	0.375
foot and mouth disease				-0.006			0.381		0.364
mouth disease				-0.026			0.314		0.360
"chicken pox"				-0.047			0.083	0.131	0.273
pristiq				-0.008			0.367		0.201
varicella									0.185

shingles				-0.081	-0.060		-0.132	0.099	0.074
cymbalta							0.294		-0.018
conjunctivitis contagious									
kalixocin									

Varicella zoster (Shingles): "Chicken pox"; clarithromycin; conjunctivitis; conjunctivitis contagious; cramping; cramps; diarrhea; discharge; kalixocin; pristiq; shingles; Varicella; white creamy discharge; white discharge

**Term**	**ACT**	**NSW**	**NT**	**Qld**	**SA**	**Tas**	**Vic**	**WA**	**Aus**
diarrhea				-0.400	0.546		0.578	0.633	0.762
cramps				-0.340	0.619		0.506	0.608	0.744
white discharge				-0.240			0.511		0.734
discharge			0.091	-0.290	0.461		0.489	0.359	0.731
cramping				-0.305			0.563		0.633
varicella									0.590
pristiq				-0.263			0.496		0.549
clarithromycin									0.444
shingles				-0.178	0.569		-0.008	0.546	0.312
conjunctivitis				-0.317			0.296		0.225
"chicken pox"				-0.409			0.111	0.521	0.097
conjunctivitis contagious									
kalixocin									
white creamy discharge									

Varicella zoster (unspecified): blood test; "Chicken pox"; clarithromycin; conjunctivitis; conjunctivitis contagious; kalixocin; pristiq; shingles; Varicella

**Term**	**ACT**	**NSW**	**NT**	**Qld**	**SA**	**Tas**	**Vic**	**WA**	**Aus**
blood test			-0.023	0.297	-0.404		0.316	0.400	0.628
pristiq				0.521			0.487		0.551
clarithromycin									0.447
varicella									0.421
conjunctivitis				0.438			0.404		0.380
shingles				0.095	-0.424		0.000	0.374	0.323
"chicken pox"				0.332			0.224	0.340	0.146
conjunctivitis contagious									
kalixocin									

Google Trends download script

```
list_of_disease = [
	// "INSERT SEARCH TERM 1",
	// "INSERT SEARCH TERM 2",
	// "INSERT SEARCH TERM 3",

]



australian_states = [
	"AU-ACT",
	"AU-NSW",
	"AU-NT",
	"AU-QLD",
	"AU-SA",
	"AU-TAS",
	"AU-VIC",
	"AU-WA",
	"AU"
]

var disease_helper = {};
DISEASE_WINDOWS_LOADED = 'windows_loaded';
DISEASE_CSV = 'csv';

// Initializing a dictionnary helper for each
// disease to remember the number of states (1 state = 1 window)
// and the csv report

var CSV_DELIMITER = ',';
var CSV_CARRIAGE = '\n';

var no_data = new Array();

var windows_loaded_callback = function(windows, disease) {
	default_state = australian_states.length - 1; // last state: the one most likely to have data
	var chartDataLength = windows[default_state].chartData.rows.length;
	for (var rowID = 0; rowID < chartDataLength; rowID++) {
		for (var k = 0; k < windows.length + 1; k++) {
			if (k === 0) {
				disease_helper[disease][DISEASE_CSV] += windows[default_state].chartData.rows[rowID][0].f + CSV_DELIMITER;
			} else {
				var temp_k = k - 1;
				try {
					disease_helper[disease][DISEASE_CSV] += windows[temp_k].chartData.rows[rowID][3];
```

```
					if (temp_k !== (windows.length - 1)) {
						disease_helper[disease][DISEASE_CSV]	+=
CSV_DELIMITER; // we dont want a , for the last value
					}
				} catch(e) {
					//console.log('Exception for temp_k:' + temp_k + ' and
rowID ' + rowID);
					disease_helper[disease][DISEASE_CSV]  += 'No Data';
					if (temp_k !== (windows.length - 1)) {
						disease_helper[disease][DISEASE_CSV]	+=
CSV_DELIMITER;
					}
				}
			}
	 }
	disease_helper[disease][DISEASE_CSV]  += CSV_CARRIAGE;
   }


	var encodedUri = encodeURI(disease_helper[disease][DISEASE_CSV]);
	var link = document.createElement("a");
	link.setAttribute("href", 'data:text/csv;charset=utf-8,' + encodedUri);
	link.setAttribute("download", disease + ".csv");
	link.click(); // This will download the data file named "my_data.csv".
}

var open_window = function(windows, current_state, current_disease, j) {
	var my_window = window.open(window.location.origin + '/trends/trendsReport?q='+
current_disease + '&geo=' + current_state + '&content=1');

	if (!my_window) {
		throw new Error('POP ups are blocked');
	}
	//TODO: if google quota exceeded, retry
	my_window.addEventListener('load', function() {
		disease_helper[current_disease][DISEASE_WINDOWS_LOADED] += 1;

		if(!this.chartData) {
			/*
			We cant find the data
			2 options here.
			  - Either there is no data from google.
			  - Or we have exceeded our quota
			*/
			if
(this.document.getElementById('report').getElementsByClassName('errorTitle')[0].innerHTM
L.indexOf('Not enough search volume') !== -1) {
				//console.log('No data for ' + current_state + ' ' +
current_disease);
```

```
                    no_data.push('No data for ' + current_state + ' ' + current_disease);
                    this.close();
                } else {
                    //PROBLEM, we ran out of quota
                    //NEED A RETRY MECHANISM
                    console.log('WARNING: LOOKS like you exceeded your quota for the data at '+ current_state + ' ' + current_disease);
                }
            } else {
                windows[j] = this;
            }

            this.close();
            if (disease_helper[current_disease][DISEASE_WINDOWS_LOADED] === australian_states.length) {
                windows_loaded_callback(windows, current_disease);
        }
   });
}

function generate() {
        disease_helper = {};
        for (var index in list_of_disease) {
            var disease = list_of_disease[index]
            disease_helper[disease] = {};
            disease_helper[disease][DISEASE_WINDOWS_LOADED] = 0;
            disease_helper[disease][DISEASE_CSV] = '';
        }

        var i = 0;
        for (var current_disease in disease_helper) {
            disease_helper[current_disease][DISEASE_CSV] += current_disease + CSV_DELIMITER;
            console.log('Processing ' + current_disease);
            for (var state_index = 0; state_index < australian_states.length; state_index++) {
                var current_state = australian_states[state_index];
                disease_helper[current_disease][DISEASE_CSV] += current_state + CSV_DELIMITER;
            }
          disease_helper[current_disease][DISEASE_CSV]  += CSV_CARRIAGE;

            var windows = new Array(australian_states.length);

            for (var j = 0; j < australian_states.length; j++) {
                var current_state = australian_states[j];

                setTimeout(function(windows, current_state, current_disease, j) {
                    return (function() {
```

```
                                        open_window(windows, current_state, current_disease,
j);
                                });
                        }(windows, current_state, current_disease, j), (i+1) * (j + 1) * 3000);
//slowing down
                }
        i++;
    }
}
```

Model construction

In this section we describe the four models that used a 52-week training period; the approach to producing the other eight models was identical but for a training period of either 104- or 156-weeks. The first model for the 52-week period was denoted 52RC (52-week model; Raw data; Continuous keyword selection). This model was built upon disease notification and search metrics data covering 52 weeks. An additional two weeks search metrics data was provided to the model upon which one and two week predictions of disease notification were made. Search metrics data used in the 52RC model were raw as they were in the format provided by Google Trends. However, the time-series for these search metrics were shifted in accordance with the 52-week cross-correlation results described above. Keyword selection was performed using a robust feature selection method based on multiple hypotheses testing and prediction from the mht R package (45). Once the predictions were recorded an additional data point was made available for each time series and the 52-week window was then shifted forward by one week. The process was then repeated using only 52-weeks data. This process allowed generation of one and two-week predictions with the linear model rebuilt for each predictions while including the most appropriate search metrics at each step.

The second model, denoted 52WC (52 week model; Wavelet transformed data; Continuous keyword selection) used an identical process to the 52RC model, except that data were denoised using wavelet transforms to be input in the linear models. The DaubLeAsymm family of wavelets from the wavethresh R package was used (46). Since the denoising is data-dependent, the wavelet-transformed data were recalculated each time the window was shifting one week because of an additional data point.

The other two models for the 52-week period (52RS: 52 week model, Raw data, Set keywords and 52WS: 52 week model, Wavelet-transformed data, Set keywords) were similarly fitted as to 52RC/52WC, except that the keyword selection in the linear models was not performed at each time point for the validation period (2012-2013). Rather, the terms selected over the training period (2009-2011) were maintained through the entire validation period. Keyword selection for these models was performed using the same process as described above for 52RC/52WC using the 2009-2011 training data. Using the 104 iterations obtained from successive shifts of the 52-week period over 2009-2011, we calculated the frequency of each selected search term in a model. The search terms selected in at least 50% of these models fitted in the training period were used for the construction of the 52RS and 52WS models.

Linear models were built on a 52-week period using the selected search terms. Prediction was obtained as described previously and the prediction accuracy was assessed based on the Mean Square Error of Prediction (provided in the supplementary material) as well as with Spearman correlation between notifications and the resulting prediction.

Models using state level Google Trends and disease notification data were also fitted using the workflow described above. Owing to the loss of Google Trends data when analysing smaller geographical areas, only models that performed well at national level were produced. Models were produced for pneumococcal disease and Ross River virus infection for Queensland, New South Wales and Victoria only.

Wavelet Transform

The search term data collected from Google Trends were pre-processed by wavelet transform. The process depends on the model, either 52WC/52WS, 104WC/WS or 156WC/WS. Since our models were tasked to predict the two following point of the disease notification, the wavelet transforms were performed on 54/106 or 158 data points, for each search term, and each time the window was moved forward one week.

Each 54/106 or 158-week period was written as the sum of weighted elementary functions, describing hierarchically the signal from a rough tendency to the finest details, in a finite number of resolution levels. Here, each signal was decomposed onto the Daubechies basis made of smooth trimodal elementary functions. The corresponding wavelet coefficients were thresholded with a soft-thresholding method (see Mallat, 1999, for details) to reduce signal noise by applying low smoothing. The original signal was then rebuilt based on the denoised wavelet coefficients.

There are several levels of decomposition of an initial spectrum from level N-1, high resolution, to level 0, rough tendency. The number of these levels depends on the number of data point; a 54-week period has N=6 levels because 54 lies between 2^5=32 and 2^6=64, a 106-week period has N=7 levels and a 158-week period has N=8 levels. The initial signal f(t) is decomposed as the sum of a detail signal D_{N-1}(t) and an approximation A_{N-1}(t). Then the approximated signal A_{N-1}(t) is decomposed into a further detail signal D_{N-2} and a further approximation A_{N-2}. Each approximated signal is decomposed sequentially as the sum of a detail signal and of an approximation signal (residual), as illustrated in Supplementary Figure 1 for the Daubechies basis. The detail signal of level *j* is obtained as:

$$D_j(t) = \sum_{k \in Z} b_{j,k}\, \Psi_{j,k}\,(t)$$

where each $\Psi_{j,k}$ is a translation and a dilation of a so-called mother wavelet $\Psi(t)$ (Daubechies trimodal function here). In practice, the index k is in a finite support. The coefficients b are called the (detailed) coefficients and are equal to $b_{j,k} = \int f(t)\Psi_{j,k}(t)dt$. An empirical estimator of these coefficients is used, from the values of the discretised signal at points t_i. Some of the numerous wavelet coefficients are close to 0, so thresholding is made to reduce the number of non-null coefficients.

Similarly, the approximated signal of level *j* is obtained as:

$$A_j(t) = \sum_{k \in Z} a_{j,k}\, \varphi_{j,k}\,(t)$$

where each $\varphi_{j,k}$ is a translation and a dilatation of a so-called father wavelet $\varphi(t)$. The coefficients a are called the approximated coefficients.

The initial signal f(t) can be entirely reconstructed from all detail signal and the approximation A0 at the lowest resolution level:

$$f(t) = A_{N-1}(t) + D_{N-1}(t) = A_0(t) + D_{N-1}(t) + D_{N-2}(t) + \cdots + D_0(t)$$

since $A_j(t) = A_{j-1}(t) + D_{j-1}(t)$ for $j \in \{N-1, N-2, \dots, 1\}$.

The (detailed) wavelet coefficients b estimated from the data in Supplementary Figure 1 are plotted in Supplementary Figure 2 for all resolution levels.

Supplementary Figure 1. Pneumococcal disease, Pneumonia search term, 106-week period starting at the tenth week of 2009 (weeks 10 to 114 to learn the model, weeks 115/116 to predict the notification).

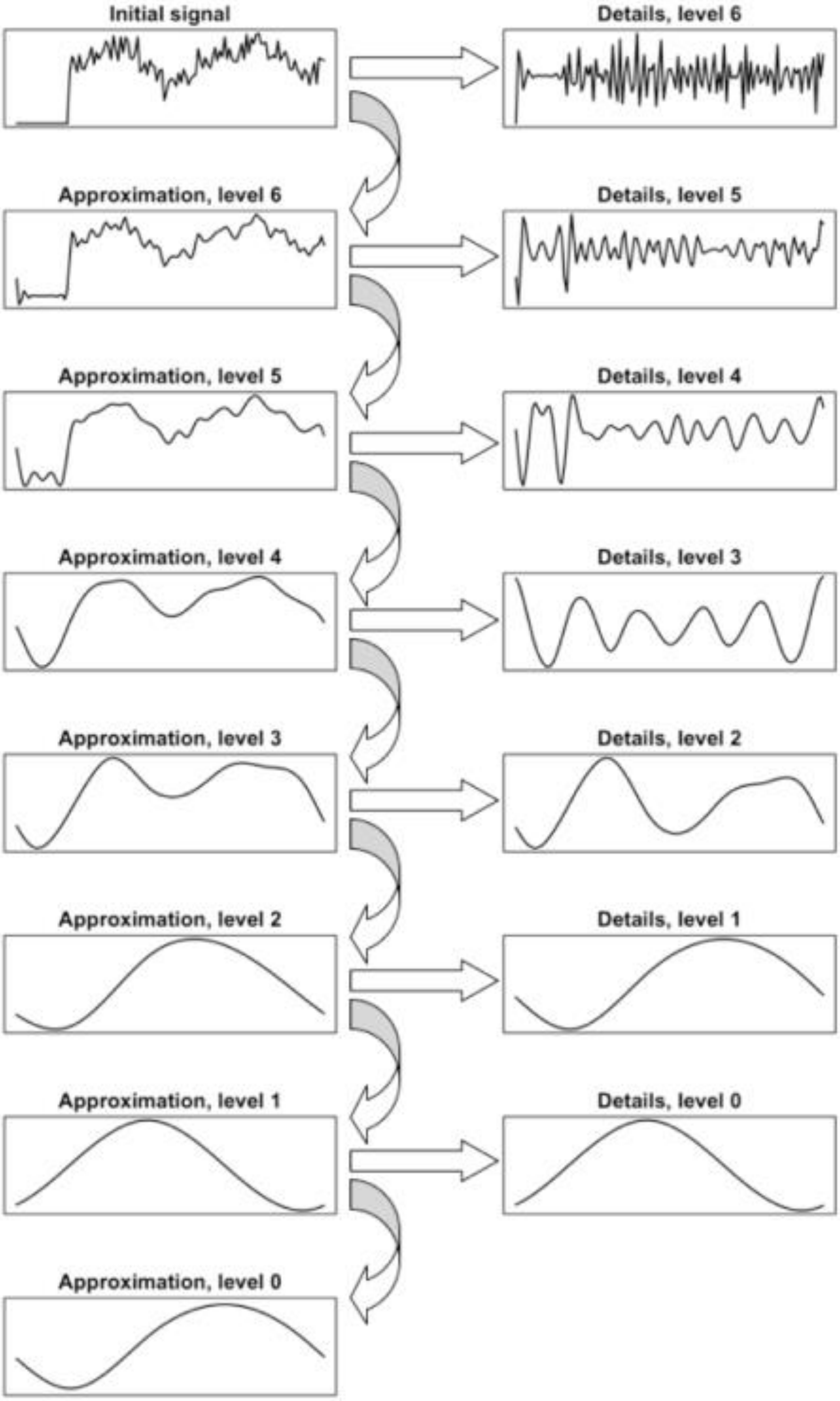

Supplementary Figure 2. Pneumococcal disease, Pneumonia search term, 106-week period starting at the tenth week of 2009 (weeks 10 to 114 to learn the model, weeks 115/116 to predict the notification).

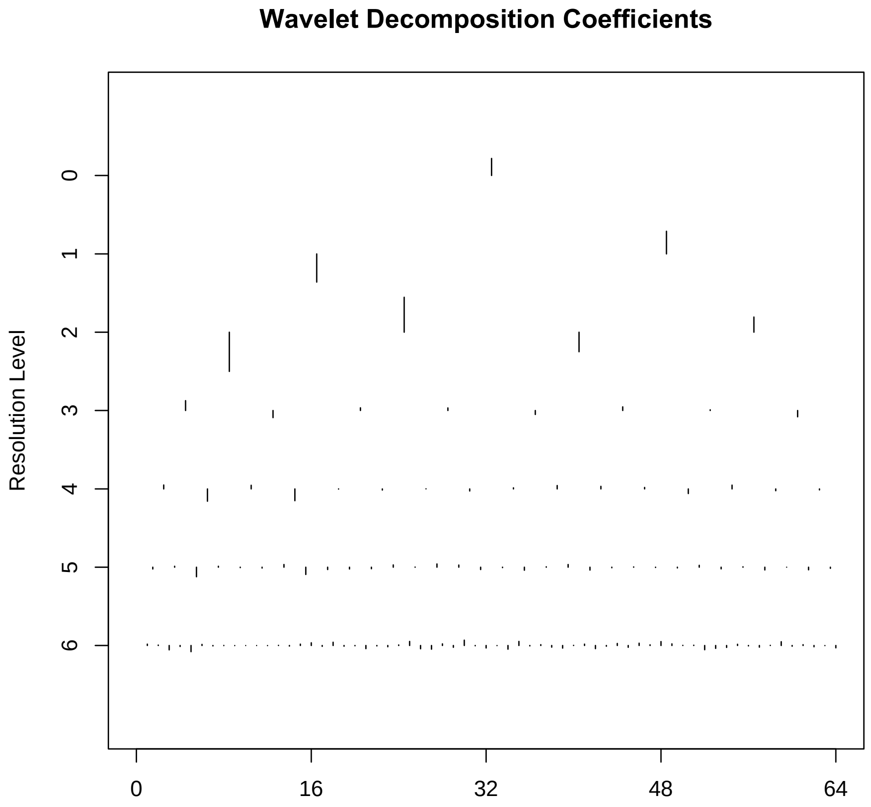

Frequency of search term selection in model training

The three tables below indicate the frequency at which each search term was selected for use in training models for pneumococcal disease, Ross River virus infection and pertussis. All values presented are proportions.

**Pneumococcal disease**	**52RC**	**52WC**	**52RS**	**52WS**	**104RC**	**104WC**	**104RS**	**104WS**	**156RC**	**156WC**	**156RS**	**156WS**
a cough	0.18	0.21	0	0	0.15	0	0	0	0	0	0	0
amoxil	0.27	0.25	0	0	0.1	0.02	0	0	0	0	0	0
bronchiolitis	0.02	0.05	0	0	0	0	0	0	0	0	0	0
bronchitis	0	0	0	0	0	0	0	0	0	0	0	0
chest infection	0.02	0.03	0	0	0	0	0	0	0	0	0	0
chesty	0.02	0.04	0	0	0.1	0.02	0	0	0	0	0	0
chesty cough	0	0.02	0	0	0.05	0.05	0	0	0	0	0	0
clarithromycin	0.22	0.24	0	0	0.02	0.02	0	0	1	1	1	1
codral	0.78	0.77	1	1	1	1	1	1	1	1	1	1
cold and flu	0.01	0.05	0	0	0	0	0	0	0	0	0	0
cold and flu tablets	0.2	0.14	0	0	0	0	0	0	0	0	0	0
cold flu	0.09	0.08	0	0	0	0.08	0	0	1	1	1	1
cold hands	0.05	0.07	0	0	0.05	0.05	0	0	0	0	0	0
cold symptoms	0.28	0.27	0	0	0.95	0.88	1	1	1	1	1	1
colds	0.11	0.11	0	0	0.1	0	0	0	0	0	0	0
cough	0.21	0.16	0	0	0	0	0	0	0	0	0	0
cough medicine	0.03	0.04	0	0	0	0	0	0	0	0	0	0
cough remedies	0.01	0.03	0	0	0.15	0.05	0	0	0	0	0	0
dry cough	0.2	0.17	0	0	0	0.02	0	0	0	0	0	0
flu tablets	0.02	0.04	0	0	0.45	0.35	0	0	0	0	0	0

is pneumonia	0.04	0.07	0	0	0.05	0.05	0	0	0	0	0	0
laryngitis	0.01	0.02	0	0	0	0	0	0	0	0	0	0
lozenges	0.09	0.14	0	0	0.12	0.05	0	0	0	0	0	0
night sweats	0	0	0	0	0	0	0	0	0	0	0	0
phenylephrine	0.36	0.32	0	0	0	0	0	0	0	0	0	0
pleurisy	0	0	0	0	0.02	0	0	0	0	0	0	0
pneumonia	0.02	0.03	0	0	0.05	0.8	0	1	1	1	1	1
red cheeks	0.02	0.03	0	0	0	0	0	0	0	0	0	0
roxithromycin	0.01	0.01	0	0	0	0	0	0	0	0	0	0
rulide	0.08	0.04	0	0	0	0	0	0	0	0	0	0
sinus	0.05	0.02	0	0	0.15	0.02	0	0	0	0	0	0
sinusitis	0	0.01	0	0	0	0	0	0	0	0	0	0
sudafed	0.35	0.15	0	0	0.02	0.02	0	0	0	0	0	0
vicks	0.07	0.03	0	0	0	0	0	0	0	0	0	0

**Ross River Virus Infection**	**52RC**	**52WC**	**52RS**	**52WS**	**104RC**	**104WC**	**104RS**	**104WS**	**156RC**	**156WC**	**156RS**	**156WS**
"Ross River"	0.41	0.36	0	0	0.98	0.98	1	1	1	1	1	1
flu injections	0	0	0	0	0	0	0	0	0	0	0	0
impetigo	0.24	0.26	0	0	0.15	0.32	0	0	1	1	1	1
ross river	0.11	0.16	0	0	0.8	0.78	1	1	1	1	1	1
ross river fever	0.42	0.41	0	0	0.18	0.38	0	0	0	0	0	0
ross river virus	0.7	0.65	1	1	0.15	0.15	0	0	0	0	0	0
school sores	0.53	0.42	0	0	0.02	0	0	0	0	0	0	0

**Pertussis**	**52RC**	**52WC**	**52RS**	**52WS**	**104RC**	**104WC**	**104RS**	**104WS**	**156RC**	**156WC**	**156RS**	**156WS**
Pertussis	0.43	0.43	0	0	0.9	0.5	1	1	1	1	1	1
whooping	0.03	0.02	0	0	0	0	0	0	0	0	0	0
whooping cough	0	0.02	0	0	0	0	0	0	0	0	0	0
whooping cough in adults	0.64	0.63	1	1	1	1	1	1	1	1	1	1

Frequency of search term selection in predictive models

The three tables below indicate the frequency at which each search term was selected for use in predictive models for pneumococcal disease, Ross River virus infection and pertussis. All values presented are proportions.

Pneumococcal disease	52RC	52WC	52RS	52WS	104RC	104WC	104RS	104WS	156RC	156WC	156RS	156WS
a cough	0.19	0.24	0	0	0.01	0.01	0	0	0.02	0	0	0
amoxil	0	0.04	0	0	0.01	0.02	0	0	0	0	0	0
bronchiolitis	0.09	0.12	0	0	0.01	0	0	0	0	0	0	0
bronchitis	0	0	0	0	0	0	0	0	0.19	0.22	0	0
chest infection	0	0.02	0	0	0	0	0	0	0.4	0.31	0	0
chesty	0.35	0.18	0	0	0.05	0.07	0	0	0	0.01	0	0
chesty cough	0.3	0.18	0	0	0.04	0.05	0	0	0.15	0.14	0	0
clarithromycin	0.07	0.05	0	0	0	0.03	0	0	0.15	0.16	1	1
codral	0.06	0.04	1	1	0.16	0.13	1	1	0.33	0.33	1	1
cold and flu	0.02	0.04	0	0	0	0.02	0	0	0.01	0.04	0	0
cold and flu tablets	0.03	0.13	0	0	0	0	0	0	0.01	0	0	0
cold flu	0.01	0.05	0	0	0.03	0.15	0	0	0.13	0.15	1	1
cold hands	0.09	0.03	0	0	0.04	0.05	0	0	0.18	0.19	0	0
cold symptoms	0.07	0.09	0	0	0.18	0.1	1	1	0.45	0.41	1	1
colds	0.18	0.27	0	0	0.05	0.01	0	0	0.01	0	0	0
cough	0.35	0.19	0	0	0.3	0.27	0	0	0.01	0.02	0	0
cough medicine	0.11	0.03	0	0	0.02	0	0	0	0	0.02	0	0
cough remedies	0.07	0.04	0	0	0.03	0	0	0	0.03	0.06	0	0
dry cough	0.26	0.15	0	0	0	0	0	0	0.02	0.02	0	0
flu tablets	0.17	0.09	0	0	0	0	0	0	0.01	0	0	0

is pneumonia	0.02	0.1	0	0	0.34	0.15	0	0	0	0.01	0	0
laryngitis	0.05	0.05	0	0	0.04	0.01	0	0	0.03	0.01	0	0
lozenges	0.25	0.31	0	0	0.04	0.03	0	0	0	0	0	0
night sweats	0.05	0.06	0	0	0.04	0	0	0	0.11	0.14	0	0
phenylephrine	0.05	0.03	0	0	0.1	0.08	0	0	0.44	0.27	0	0
pleurisy	0.27	0.05	0	0	0.26	0.1	0	0	0	0	0	0
pneumonia	0	0.12	0	0	0.76	0.77	0	1	0.93	0.94	1	1
red cheeks	0.06	0.06	0	0	0	0	0	0	0	0	0	0
roxithromycin	0.07	0.15	0	0	0	0.01	0	0	0.04	0.04	0	0
rulide	0.04	0.05	0	0	0.4	0.22	0	0	0.2	0.17	0	0
sinus	0.02	0.13	0	0	0.2	0.09	0	0	0.04	0.05	0	0
sinusitis	0.07	0.1	0	0	0	0	0	0	0.04	0.01	0	0
sudafed	0.77	0.54	0	0	0.77	0.65	0	0	0.79	0.76	0	0
vicks	0	0.04	0	0	0	0	0	0	0.14	0.08	0	0

**Ross River Virus Infection**	**52RC**	**52WC**	**52RS**	**52WS**	**104RC**	**104WC**	**104RS**	**104WS**	**156RC**	**156WC**	**156RS**	**156WS**
"Ross River"	0.94	0.75	0	0	1	0.95	1	1	1	1	1	1
flu injections	0	0	0	0	0	0	0	0	0	0	0	0
impetigo	0.06	0.13	0	0	0.15	0.25	0	0	1	1	1	1
ross river	0.03	0.28	0	0	1	1	1	1	1	1	1	1
ross river fever	0.13	0.1	0	0	0.12	0.12	0	0	0.12	0.1	0	0
ross river virus	0.74	0.7	1	1	0.01	0.04	0	0	0	0	0	0
school sores	0.34	0.23	0	0	0.43	0.12	0	0	0	0	0	0

**Pertussis**	**52RC**	**52WC**	**52RS**	**52WS**	**104RC**	**104WC**	**104RS**	**104WS**	**156RC**	**156WC**	**156RS**	**156WS**
Pertussis	0.39	0.39	0	0	0.35	0.2	1	1	0.01	0.01	1	1
whooping	0.03	0.02	0	0	0	0	0	0	0	0	0	0
whooping cough	0	0.02	0	0	0	0	0	0	0	0	0	0
whooping cough in adults	0.58	0.57	1	1	0.39	0.39	1	1	0.01	0.01	1	1

National model performance – Spearman's correlation

National model performance for 1 week (top) and 2 week estimates (bottom), as assessed by Spearman's correlation. The highest performing models for each disease are indicated in bold.

1 Week estimate

	52RC	*52WC*	*52RS*	*52WS*	*104RC*	*104WC*	*104RS*	*104WS*	*156RC*	*156WC*	*156RS*	*156WS*
Gonococcal infection	0.026	0.038	0.028	0.025	**0.088**	0.064	0.059	0.067	0.072	0.043	0.047	0.044
Varicella zoster (Shingles)	0.089	0.088	**0.180**	0.173	0.110	0.088	0.099	0.115	0.061	0.045	0.080	0.075
Pneumococcal disease (invasive)	0.602	0.592	0.567	0.493	**0.742**	0.693	0.671	0.689	0.703	0.700	0.693	0.678
Ross River virus infection	0.700	0.680	0.648	0.687	**0.741**	0.684	0.720	0.703	0.728	0.731	0.729	0.732
Pertussis	0.594	**0.597**	0.593	0.594	0.330	0.387	0.347	0.221	0.213	0.208	0.276	0.269
Chlamydial infection	0.000	0.002	0.008	0.004	0.119	**0.120**	0.119	0.120	0.097	0.100	0.097	0.100
Varicella zoster (unspecified)	0.353	**0.403**	0.336	0.290	0.297	0.260	0.340	0.327	0.301	0.338	0.291	0.291
Varicella zoster (Chickenpox)	0.232	0.234	**0.357**	**0.357**	0.305	0.325	0.350	**0.357**	0.199	0.158	0.223	0.207
Cryptosporidiosis			0.245	**0.324**			0.251	0.307			0.314	0.313
Barmah Forest virus infection	0.246	0.217	**0.303**	0.177	0.174	0.176	0.161	0.140	0.186	0.165	0.167	0.162
Dengue virus infection	0.002	0.004	0.003	0.000	0.000	0.006	0.003	0.006	0.004	**0.009**	0.003	0.003
Influenza (laboratory confirmed)	0.327	0.317	0.326	0.322	**0.401**	0.316	0.356	0.290	0.274	0.283	0.236	0.219

2 Week estimate

	52RC	*52WC*	*52RS*	*52WS*	*104RC*	*104WC*	*104RS*	*104WS*	*156RC*	*156WC*	*156RS*	*156WS*
Gonococcal infection	0.024	0.037	0.028	0.009	0.054	0.026	0.051	**0.061**	0.060	0.044	0.047	0.051
Varicella zoster (Shingles)	0.071	0.075	**0.175**	0.163	0.126	0.086	0.100	0.089	0.088	0.024	0.074	0.059
Pneumococcal disease (invasive)	0.674	0.617	0.571	0.542	**0.734**	0.711	0.673	0.705	0.698	0.688	0.695	0.681
Ross River virus infection	0.680	0.705	0.669	0.693	0.736	0.718	0.719	**0.749**	0.723	0.729	0.723	0.729
Pertussis	0.573	**0.583**	0.581	0.579	0.312	0.321	0.321	0.203	0.205	0.213	0.272	0.275
Chlamydial infection	0.000	0.000	0.002	0.005	**0.106**	0.085	**0.106**	0.085	0.081	0.099	0.081	0.099
Varicella zoster (unspecified)	0.298	0.283	0.354	0.346	0.329	0.275	0.340	0.339	0.350	**0.368**	0.316	0.318
Varicella zoster (Chickenpox)	0.263	**0.374**	0.357	0.361	0.260	0.269	0.344	0.371	0.143	0.116	0.188	0.183
Cryptosporidiosis			0.235	**0.326**			0.242	0.307			0.293	0.284
Barmah Forest virus infection	0.217	0.205	**0.260**	0.151	0.144	0.125	0.145	0.125	0.154	0.150	0.144	0.141
Dengue virus infection	0.001	0.000	0.005	0.001	0.002	0.002	0.000	**0.006**	0.000	0.003	0.005	0.003
Influenza (laboratory confirmed)	0.317	0.298	0.313	0.325	**0.381**	0.345	0.345	0.303	0.231	0.238	0.192	0.199

National model performance – Mean Square Error of Prediction

National model performance for 1 week (top) and 2 week estimates (bottom), as assessed by Mean Square Error of Prediction. The highest performing models for each disease are indicated in bold.

1 Week estimate

	52RC	52WC	52RS	52WS	104RC	104WC	104RS	104WS	156RC	156WC	156RS	156WS
Gonococcal infection	1.183	1.237	1.212	1.229	**1.181**	1.301	1.274	1.368	1.268	1.34	1.356	1.358
Varicella zoster (Shingles)	0.97	1.105	**0.84**	0.85	0.907	0.964	0.946	0.937	0.954	1.008	0.967	0.976
Pneumococcal disease (invasive)	0.478	0.51	0.523	0.548	**0.278**	0.347	0.564	0.376	0.42	0.396	0.435	0.437
Ross River virus infection	0.394	0.542	0.465	0.514	0.365	0.537	0.351	0.478	0.289	**0.288**	0.29	**0.288**
Pertussis	1.418	**1.394**	1.447	1.416	2.053	1.659	2.206	2.118	2.299	2.265	2.24	2.189
Chlamydial infection	1.089	1.019	0.983	0.968	0.832	**0.789**	0.832	**0.789**	0.847	0.826	0.847	0.826
Varicella zoster (unspecified)	0.771	**0.764**	0.83	0.845	0.892	0.942	0.883	0.909	0.994	0.957	1.014	1.014
Varicella zoster (Chickenpox)	0.862	0.879	**0.746**	0.758	0.773	0.805	0.76	0.76	0.875	0.926	0.884	0.861
Cryptosporidiosis			**1.031**	1.05			1.071	1.089			1.048	1.049
Barmah Forest virus infection	1.527	1.52	**1.072**	1.249	1.188	1.214	1.201	1.267	1.18	1.189	1.197	1.203
Dengue virus infection	1.362	1.748	1.355	1.417	1.22	1.263	1.149	1.177	**1.101**	1.11	1.334	1.342
Influenza (laboratory confirmed)	0.425	0.463	**0.406**	0.438	0.461	0.485	0.461	0.49	0.718	0.681	0.915	0.922

2 Week estimate

	52RC	52WC	52RS	52WS	104RC	104WC	104RS	104WS	156RC	156WC	156RS	156WS
Gonococcal infection	1.213	**1.145**	1.24	1.217	1.231	1.235	1.306	1.181	1.317	1.266	1.367	1.267
Varicella zoster (Shingles)	1.015	1.071	0.846	**0.82**	0.903	0.907	0.95	0.918	0.918	1.02	0.979	0.966
Pneumococcal disease (invasive)	0.388	0.496	0.529	0.473	**0.311**	0.374	0.577	0.454	0.403	0.462	0.446	0.495
Ross River virus infection	0.425	0.449	0.439	0.452	0.364	0.465	0.35	0.378	**0.293**	0.303	0.295	0.304
Pertussis	1.575	**1.562**	1.594	1.571	2.166	1.922	2.399	2.313	2.453	2.436	2.397	2.378
Chlamydial infection	1.052	1.103	0.992	1.06	**0.856**	0.991	**0.856**	0.991	0.884	0.948	0.884	0.948
Varicella zoster (unspecified)	0.886	**0.813**	0.869	0.878	0.951	0.854	0.929	0.854	1.034	0.98	1.046	0.995
Varicella zoster (Chickenpox)	0.862	0.771	**0.768**	0.789	0.832	0.93	0.769	0.781	0.969	1.06	0.923	0.908
Cryptosporidiosis			1.092	1.098			1.089	1.096			**1.058**	**1.058**
Barmah Forest virus infection	1.643	1.677	**1.129**	1.257	1.202	1.265	1.221	1.249	1.204	1.2	1.221	1.222
Dengue virus infection	1.373	1.527	1.335	1.344	1.269	1.277	1.178	1.175	1.172	**1.147**	1.341	1.309
Influenza (laboratory confirmed)	0.473	0.464	0.428	**0.42**	0.49	0.473	0.501	0.481	0.769	0.734	0.966	0.966

State model performance

State model performance for 1 week (top) and 2 week estimates (bottom), as assessed by Spearman's rank correlation. The highest performing models for each disease are indicated in bold. All values presented are Spearman rho.

1 Week estimate												
**Pneumococcal disease (invasive)**	*52RC*	*52WC*	*52RS*	*52WS*	*104RC*	*104WC*	*104RS*	*104WS*	*156RC*	*156WC*	*156RS*	*156WS*
Australia	0.602	0.592	0.567	0.493	**0.742**	0.693	0.671	0.689	0.703	0.700	0.693	0.678
New South Wales	0.345	0.196	0.342	0.348	0.440	0.362	0.381	0.423	0.462	**0.469**	0.444	0.447
Queensland	0.306	0.320	0.310	0.233	0.286	0.263	0.304	0.294	0.326	0.332	**0.344**	0.342
Victoria	0.402	0.309	0.039	0.039	0.342	0.341	0.401	0.288	0.409	**0.416**	0.362	0.383
**Ross River virus infection**	*52RC*	*52WC*	*52RS*	*52WS*	*104RC*	*104WC*	*104RS*	*104WS*	*156RC*	*156WC*	*156RS*	*156WS*
Australia	0.700	0.680	0.648	0.687	**0.741**	0.684	0.720	0.703	0.728	0.731	0.729	0.732
New South Wales			0.162	0.126			0.214	0.209			0.219	**0.239**
Queensland			0.298	**0.362**			0.264	0.197			0.200	0.197
Victoria												

2 Week estimate												
**Pneumococcal disease (invasive)**	*52RC*	*52WC*	*52RS*	*52WS*	*104RC*	*104WC*	*104RS*	*104WS*	*156RC*	*156WC*	*156RS*	*156WS*
Australia	0.674	0.617	0.571	0.542	**0.734**	0.711	0.673	0.705	0.698	0.688	0.695	0.681
New South Wales	0.289	0.259	0.333	0.358	0.407	**0.459**	0.376	0.427	0.436	0.453	0.426	0.418
Queensland	0.232	0.235	0.307	0.258	0.266	0.253	0.309	0.315	**0.370**	0.339	0.346	0.356
Victoria	0.342	0.367	0.055	0.055	0.319	0.318	**0.389**	0.309	0.386	0.370	0.354	0.369
**Ross River virus infection**	*52RC*	*52WC*	*52RS*	*52WS*	*104RC*	*104WC*	*104RS*	*104WS*	*156RC*	*156WC*	*156RS*	*156WS*
Australia	0.680	0.705	0.669	0.693	0.736	0.718	0.719	**0.749**	0.723	0.729	0.723	0.729
New South Wales			0.173	0.135			0.236	0.237			0.236	**0.251**
Queensland			0.288	**0.363**			0.219	0.152			0.141	0.142
Victoria												

National model performance - Mean Square Error of Prediction

National model performance for 1 week (top) and 2 week estimates (bottom), as assessed by Mean Square Error of Prediction. The highest performing models for each disease are indicated in bold.

**1 Week estimate**												
	52RC	***52WC***	***52RS***	***52WS***	***104RC***	***104WC***	***104RS***	***104WS***	***156RC***	***156WC***	***156RS***	***156WS***
Gonococcal infection	1.183	1.237	1.212	1.229	**1.181**	1.301	1.274	1.368	1.268	1.340	1.356	1.358
Varicella zoster (Shingles)	0.970	1.105	**0.840**	0.850	0.907	0.964	0.946	0.937	0.954	1.008	0.967	0.976
Pneumococcal disease (invasive)	0.478	0.510	0.523	0.548	**0.278**	0.347	0.564	0.376	0.420	0.396	0.435	0.437
Ross River virus infection	0.394	0.542	0.465	0.514	0.365	0.537	0.351	0.478	0.289	**0.288**	0.290	**0.288**
Pertussis	1.418	**1.394**	1.447	1.416	2.053	1.659	2.206	2.118	2.299	2.265	2.240	2.189
Chlamydial infection	1.089	1.019	0.983	0.968	0.832	**0.789**	0.832	**0.789**	0.847	0.826	0.847	0.826
Varicella zoster (unspecified)	0.771	**0.764**	0.830	0.845	0.892	0.942	0.883	0.909	0.994	0.957	1.014	1.014
Varicella zoster (Chickenpox)	0.862	0.879	**0.746**	0.758	0.773	0.805	0.760	0.760	0.875	0.926	0.884	0.861
Cryptosporidiosis			**1.031**	1.050			1.071	1.089			1.048	1.049
Barmah Forest virus infection	1.527	1.520	**1.072**	1.249	1.188	1.214	1.201	1.267	1.180	1.189	1.197	1.203
Dengue virus infection	1.362	1.748	1.355	1.417	1.220	1.263	1.149	1.177	**1.101**	1.110	1.334	1.342
Influenza (laboratory confirmed)	0.425	0.463	**0.406**	0.438	0.461	0.485	0.461	0.490	0.718	0.681	0.915	0.922

**2 Week estimate**												
	52RC	***52WC***	***52RS***	***52WS***	***104RC***	***104WC***	***104RS***	***104WS***	***156RC***	***156WC***	***156RS***	***156WS***
Gonococcal infection	1.213	**1.145**	1.240	1.217	1.231	1.235	1.306	1.181	1.317	1.266	1.367	1.267
Varicella zoster (Shingles)	1.015	1.071	0.846	**0.820**	0.903	0.907	0.950	0.918	0.918	1.020	0.979	0.966
Pneumococcal disease (invasive)	0.388	0.496	0.529	0.473	**0.311**	0.374	0.577	0.454	0.403	0.462	0.446	0.495
Ross River virus infection	0.425	0.449	0.439	0.452	0.364	0.465	0.350	0.378	**0.293**	0.303	0.295	0.304
Pertussis	1.575	**1.562**	1.594	1.571	2.166	1.922	2.399	2.313	2.453	2.436	2.397	2.378
Chlamydial infection	1.052	1.103	0.992	1.060	**0.856**	0.991	**0.856**	0.991	0.884	0.948	0.884	0.948
Varicella zoster (unspecified)	0.886	**0.813**	0.869	0.878	0.951	0.854	0.929	0.854	1.034	0.980	1.046	0.995

Varicella zoster (Chickenpox)	0.862	0.771	**0.768**	0.789	0.832	0.930	0.769	0.781	0.969	1.060	0.923	0.908
Cryptosporidiosis			1.092	1.098			1.089	1.096			**1.058**	**1.058**
Barmah Forest virus infection	1.643	1.677	**1.129**	1.257	1.202	1.265	1.221	1.249	1.204	1.200	1.221	1.222
Dengue virus infection	1.373	1.527	1.335	1.344	1.269	1.277	1.178	1.175	1.172	**1.147**	1.341	1.309
Influenza (laboratory confirmed)	0.473	0.464	0.428	**0.420**	0.490	0.473	0.501	0.481	0.769	0.734	0.966	0.966
